# Antibody Specificity Following a Recent *Bordetella pertussis* Infection in Adolescence Is Correlated With the Pertussis Vaccine Received in Childhood

**DOI:** 10.3389/fimmu.2019.01364

**Published:** 2019-06-17

**Authors:** René H. M. Raeven, Larissa van der Maas, Jeroen L. A. Pennings, Kurt Fuursted, Charlotte Sværke Jørgensen, Elly van Riet, Bernard Metz, Gideon F. A. Kersten, Tine Dalby

**Affiliations:** ^1^Intravacc (Institute for Translational Vaccinology), Bilthoven, Netherlands; ^2^Centre for Health Protection, National Institute for Public Health and the Environment, Bilthoven, Netherlands; ^3^Statens Serum Institut, Infectious Disease Preparedness, Copenhagen, Denmark; ^4^Leiden Academic Center for Drug Research, Division of Biotherapeutics, Leiden University, Leiden, Netherlands

**Keywords:** *Bordetella pertussis*, vaccination, acellular pertussis vaccine, whole-cell pertussis vaccine, 2-dimensional electrophoresis (2DE), antibody specificity, infection-induced response, pertussis toxin (PTx)

## Abstract

*Bordetella (B.) pertussis* resurgence affects not only the unvaccinated, but also the vaccinated population. Different vaccines are available, however, it is currently unknown whether the type of childhood vaccination has an influence on antibody responses following a *B. pertussis* infection later in life. Therefore, the study aim was to profile serum antibody responses in young adults with suspected *B. pertussis* infections, immunized during childhood with either whole-cell (wPV) or monocomponent acellular pertussis (aPV) vaccines. Serum anti-pertussis toxin (PTx) IgG antibody levels served as an indicator for a recent *B. pertussis* infection. Leftover sera from a diagnostic laboratory from 36 Danish individuals were included and divided into four groups based on immunization background (aPV *vs*. wPV) and serum anti-PTx IgG levels (– *vs*. +). Pertussis-specific IgG/IgA antibody levels and antigen specificity were determined by using multiplex immunoassays (MIA), one- and two-dimensional immunoblotting (1 & 2DEWB), and mass spectrometry. Besides enhanced anti-PTx levels, wPV(+) and aPV(+) groups showed increased IgG and IgA levels against pertactin, filamentous hemagglutinin, fimbriae 2/3, and pertussis outer membrane vesicles (OMV). In the wPV(–) and aPV(–) groups, only low levels of anti-OMV antibodies were detected. 1DEWB demonstrated that antibody patterns differed between groups but also between individuals with the same immunization background and anti-PTx levels. 2DWB analysis for serum IgG revealed 133 immunogenic antigens of which 40 were significantly different between groups allowing to differentiate wPV(+) and aPV(+) groups. Similarly, for serum IgA, 7 of 47 immunogenic protein spots were significantly different. This study demonstrated that *B. pertussis* infection-induced antibody responses were distinct on antigen level between individuals with either wPV or aPV immunization background. Importantly, only 2DEWB and not MIA could detect these differences indicating the potential of this method. Moreover, in individuals immunized with an aPV containing only PTx in childhood, the infection-induced antibody responses were not limited to PTx alone.

## Introduction

Pertussis vaccines have been in use since the middle of the twentieth century and have successfully decreased the incidence of pertussis. However, despite worldwide pertussis immunizations, an increase in pertussis cases has been observed both in developing countries that are using inactivated whole-cell pertussis vaccines (wPV) as well as in industrialized countries that mainly use acellular pertussis vaccines (aPV) (1). Comparisons of incidences between countries are however virtually impossible (2). The observed increases in pertussis are thought to be a combination of improved surveillance, improved diagnostic methods, the emergence of more virulent *Bordetella (B.) pertussis* strains as well as rapid waning of aPV-induced immunity and the failure of current pertussis vaccines to prevent infection and transmission on the longer term (3, 4).

Neither infection, nor any pertussis vaccine to date can confer life-long protection against *B. pertussis*. However, several different vaccine formulations are on the market that induce different types of immune responses. The composition of multi-component wPVs fluctuates between producers, and also the available aPVs vary in the type, number, and concentration of antigen components. For instance in Denmark, the wPV was replaced in 1997 by a monocomponent aPV that solely contains pertussis toxin (5). Antibody responses to pertussis toxoid and pertussis toxin (PTx) are protective (6) and are used to monitor pertussis vaccine- and *B. pertussis* infection-induced responses (7). Anti-PTx antibodies alone have been shown to be sufficient to protect against pertussis disease (8, 9).

Vaccine-induced antibody responses wane within 1–2 years post-immunization (10, 11). However, little is known about the differences in immune-response to subsequent *B. pertussis* (re)infection years later (12). Moreover, it remains unknown whether the vaccine given at childhood has an influence on an infection-induced immune response in the years after the expected protection has run out. Previously, it was claimed that immune-responses to later *B. pertussis* infection would be targeted at vaccine-antigens only and not to the full spectrum of *B. pertussis* antigens, the so-called “original antigenic sin” (13), and “linked epitope suppression” (14), potentially leading to a skewed and impaired antibody-response. Whether a distinct childhood immunization program influences antibody responses following *B. pertussis* reinfection several years later in life requires an in-depth serological investigation.

*B. pertussis* infection-induced humoral responses have been investigated in the past using enzyme-linked immunosorbent assays (ELISA) or multiplex immunoassays (MIA) (11, 12, 15–17). However, in order to detect differences in the complex and diverse antibody responses following a *B. pertussis* infection, a broader approach is required. Valentini et al. applied peptide microarray analysis to detect differences in serum antibody recognition patterns induced by *B. pertussis* infection and different pertussis vaccines (18). Furthermore, a combination of two-dimensional electrophoresis (2DE) with Western blotting was previously used for immunoproteomic profiling of pertussis vaccine-induced and *B. pertussis* infection-induced antibodies in mice (19, 20) as well as analysis of human serological antibody responses to wPV vaccination (21). These studies demonstrated a higher resolution of the antibody response-patterns and therefore, an added value of the application of 2DE for the unraveling of antibody profiles in pertussis research.

In this study, we performed an in-depth serological investigation of the antibody repertoire following a *B. pertussis* infection in early adulthood looking at the dependence of vaccine priming (wPV or a monovalent aPV) in infancy. To that end, antigen specificity of IgG and IgA antibodies in sera of 36 Danish individuals that were either vaccinated with aPV or wPV, was analyzed by MIA, gel electrophoresis, immunoblotting, and mass spectrometry (MS).

## Materials and Methods

### Sample Description

Left-over material of serum submitted for diagnostic analysis in the period 2014 to 2016 at Statens Serum Institut, Copenhagen, Denmark was used. The sera had all been submitted for diagnosis of pertussis by analysis of anti-PTx IgG (22). Information on the sera is shown in Table 1. There are no clinical data available from the individuals from whom the sera originated. Symptoms or length of disease at the time of sampling are therefore unknown. The study was approved by the National Committee on Health Research Ethics, Denmark (H-16037386).

**Table 1:** Sample information of the included individuals and type of analysis performed on the serum.

**Sample information**										**Analysis**					
**Group ID**	**#**	**Sample ID**	**Sample year**	**Sex**	**Year of birth**	**Age [years]**	**Anti-PTx IgG [IU/ml]**	**Vaccination scheme (months/years)**	**Years since vaccination**	**MIA**		**SDS-PAGE**		**2DEWB**	
										**IgG**	**IgA**	**IgG**	**IgA**	**IgG**	**IgA**
	1	SSI-05	2016	Female	1999	16	6	3, 5, 12 m + 5 y	12	X	X	X	X		X
	2	SSI-08	2016	Male	1998	17	6	3, 5, 12 m + 5 y	11	X	X	X	X		
	3	SSI-25	2016	Female	1999	17	4	3, 5, 12 m + 5 y	12	X	X	X	X		
	4	SSI-26	2016	Female	1999	16	12	3, 5, 12 m + 5 y	11	X	X	X	X	X	
aPV low anti-PTx (aPV-)	5	SSI-27	2015	Female	1998	17	13	3, 5, 12 m + 5 y	12	X	X	X	X		
	6	SSI-28	2015	Female	1999	15	8	3, 5, 12 m + 5 y	11	X	X	X	X	X	
	7	SSI-29	2015	Female	1998	16	9	3, 5, 12 m + 5 y	12	X	X	X	X	X	
	8	SSI-34	2015	Female	2000	15	3	3, 5, 12 m + 5 y	10	X	X	X	X	X	X
	9	SSI-35	2016	Female	2000	15	15	3, 5, 12 m + 5 y	10	X	X	X	X		
Mean ± Stdev						16.0 ± 0.8									
	10	SSI-03	2016	Female	1994	21	3			X	X	X	X	X	X
	11	SSI-04	2016	Male	1994	22	3			X	X	X	X		
	12	SSI-13	2016	Female	1995	21	12			X	X	X	X		
	13	SSI-14	2016	Female	1995	20	3			X	X	X	X		
wPV low anti-PTx (wPV-)	14	SSI-15	2015	Female	1995	20	14			X	X	X	X		
	15	SSI-16	2015	Male	1995	20	14			X	X	X	X	X	
	16	SSI-17	2015	Female	1995	20	9			X	X	X	X	X	X
	17	SSI-18	2015	Female	1995	20	9			X	X	X	X	X	
	18	SSI-19	2015	Male	1995	20	4			X	X	X	X		
Mean ± Stdev						20.4 ± 0.7									
	19	SSI-02	2016	Male	1999	16	≥260	3, 5, 12 m + 5 y	11	X	X	X	X	X	X
	20	SSI-06	2015	Male	1999	15	203	3, 5, 12 m + 5 y	10	X	X	X	X	X	X
	21	SSI-20	2016	Female	1999	17	≥260	3, 5, 12 m + 5 y	12	X	X	X	X		
	22	SSI-21	2016	Female	1999	16	229	3, 5, 12 m + 5 y	12	X	X	X	X		
aPV high anti-PTx (aPV+)	23	SSI-22	2015	Male	1999	15	≥260	3, 5, 12 m + 5 y	11	X	X	X	X		
	24	SSI-23	2015	Female	1999	16	≥260	3, 5, 12 m + 5 y	11	X	X	X	X		
	25	SSI-24	2015	Male	1999	15	≥260	3, 5, 12 m + 5 y	10	X	X	X	X	X	
	26	SSI-32	2014	Female	1999	14	182	3, 5, 12 m + 5 y	10	X	X	X	X	X	
	27	SSI-33	2014	Female	1998	15	≥260	3, 5, 12 m + 5 y	11	X	X	X	X		
Mean ± Stdev						15.4 ± 0.8									
	28	SSI-01	2015	Male	1995	19	≥260			X	X	X	X		X
	29	SSI-07	2015	Male	1995	20	151			X	X	X	X	X	X
	30	SSI-09	2016	Female	1995	21	196			X	X	X	X		
	31	SSI-10	2016	Female	1995	20	≥260			X	X	X	X	X	
wPV high anti-PTx (wPV+)	32	SSI-11	2016	Female	1995	20	187			X	X	X	X		
	33	SSI-12	2016	Female	1995	20	≥260			X	X	X	X		
	34	SSI-30	2014	Male	1994	19	≥260			X	X	X	X		
	35	SSI-31	2014	Male	1995	19	≥260			X	X	X	X	X	
	36	SSI-36	2016	Male	1994	21	≥260			X	X	X	X	X	
Mean ± Stdev						19.9 ± 0.7									

The pertussis vaccine used for the primary series in Denmark changed in 1997 from a whole-cell to an acellular monocomponent with pertussis toxoid as the sole pertussis antigen. Sera were chosen from individuals from the wPV period and the aPV period in equal numbers. In order to minimize any differences between the groups due to age, the sera were selected from individuals born just a few years prior (wPV group) or after (aPV group) 1997. To increase the discriminatory power of the study, sera were chosen to have either very low ( ≤ 15 IU/ml, the -group) or very high anti-PTx IgG levels (≥150 IU/ml, the +group) in equal numbers. A total of 36 sera were chosen, nine in each group designated as: wPV(+), wPV(–), aPV(+), and aPV(–).

By using the Danish vaccination register (23), information on received pertussis immunizations were collected for the individuals from whom the sera originated. The register only holds information from 1996 and onwards, and information on immunizations is therefore, only available for the sera from individuals in the aPV groups. Therefore, while the aPV background is certain, the wPV background is only assumed, but the vaccination coverage in Denmark has been high throughout the period. Sera from the aPV group were selected to originate from individuals who have received the full 3 + 5 + 12 months + 5 years vaccination series. The latest received vaccination was thus ~10 to 12 years prior to the serum sample being drawn, i.e., after the presumed duration of immunity of the vaccine. Sera from the wPV group were chosen from individuals with no registrations of having received an aPV, neither infant series nor booster. There are no other recommendations for pertussis-vaccinations in Denmark after the 5 year booster.

The vaccines used in Denmark in the period where the study individuals were vaccinated were: infant series with a stand-alone wPV (Statens Serum Institut, Denmark) until December 1996, infant series with the acellular *DiTeKiPol* (Statens Serum Institut, Denmark) from January 1997 and a pre-school booster at 5 years of age with *diTekiBooster* (Statens Serum Institut, Denmark) from September 2003.

### Multiplex Immunoassay (MIA)

The levels of total IgA and IgG against filamentous hemagglutinin (FHA) (Sigma Aldrich, Germany), combined fimbriae type 2 and 3 antigens (Fim2/3), pertactin (Prn), pertussis toxin (PTx) (Biotrend, Germany), and outer membrane vesicles (OMV) of *B. pertussis* strain B1917 (OMV B1917) in human sera were determined using MIA. Conjugation of OMVs and purified antigens to beads was performed as described previously (24). Sera were diluted, 1,000-fold for total IgG and 100-fold for IgA, in PBS (Thermo Fisher, Gibco, Netherlands) containing 0.1% Tween-20 (Sigma Aldrich, Merck, Germany) and 3% bovine serum albumin (Sigma Aldrich, Merck, Germany). The diluted sera were mixed 1 + 1 with 25 μl conjugated beads (4,000 beads/(region/well)) and incubated for 30–45 min at RT at 600 rpm. Subsequently, samples were incubated with R-Phycoerythrin (RPE)-conjugated anti-human IgA (1:100) and IgG (1:1,000) and analyzed using the Bio-Plex System (Bio-Plex 200, BioRad, Netherlands). Samples were analyzed in singlets of two different dilutions of which one was used for further analysis. Antibody level results were depicted with GraphPad Prism 7.01 (GraphPad Software, Inc.), and presented in fluorescent intensity (F.I.).

### Sample Preparation for SDS-PAGE and 2D Electrophoresis

*Bordetella pertussis* B1917 was cultivated as described previously (25) to obtain a bacterial lysate in the virulent state. Subsequently, the bacteria were heat-inactivated at 56°C for 30 min. Of this lysate, 500 μl was centrifuged (30 min, 17,000 g) (Heraeus™ Pico™ 17 Microcentrifuge, Thermo Fisher, Netherlands). Supernatant was discarded and pellet was stored overnight at −80°C. Pellet was resolved in 500 μl Bacterial Protein Extraction Reagent (BPER) (Thermo Fisher, Netherlands). Protein concentration was determined using a Bicinchonicic acid (BCA) assay (Thermo Fisher, Pierce, Netherlands). The antigen composition of this lysate has been extensively investigated previously with LC-MS (19).

### Infrared Labeling

IR800 label (100 μg) (Licor, Westburg, Netherlands) was dissolved in 25 μl distilled water. Subsequently, IR800 label was conjugated to secondary antibodies by mixing 100 μl goat-anti-human IgA (Southern Biotech, USA, Alabama) or rabbit-anti-human IgG (Thermo Fisher, Netherlands) with 1 or 2 μl IR800 label, respectively. Unbound IR label was removed from the samples using ZEBA spin desalting column (Thermo Fisher, Netherlands).

### SDS PAGE

An amount of 10 μg *B. pertussis* B1917 lysate (see sample preparation) was incubated with 4 × reducing sample buffer (250 mM Tris (Thermo Fisher, Merck, Netherlands), 8% SDS (Sigma Aldrich, Germany), 400 mM DTT (Sigma Aldrich, Germany), 40% Glycerol (Sigma Aldrich, Germany), and 0.04% bromophenol blue (Sigma Aldrich, Germany) 10 min at 100°C. Sample was loaded on 10% NuPAGE bis tris 1.0 mm precast gel (Thermo Fisher, Invitrogen, Netherlands) and proteins were separated with MES running buffer (Thermo Fisher, Invitrogen, Netherlands), 200 Volt (V) for 45 min in Xcell surelock minicell system (Thermo Fisher, Invitrogen, Netherlands). Gel was either stained with Coomassie (Imperial protein stain, Thermo Fisher, Netherlands) or used for Western blot.

### 2D Electrophoresis

After the sample preparation, 50 μg *B. pertussis* B1917 lysate was incubated for 30 min at RT after adding 10 μl 250 mM DTT, 0.62 μl IPG buffer pH 3–10 non-linear (NL), or IPG buffer pH 4-7 (GE Healthcare, Netherlands), and DeStreak Rehydration Solution (GE Healthcare, Netherlands) to a final volume of 115 μl. Immobiline DryStrips pH 3–10 NL, 7 cm, or pH 4–7, 7 cm (GE Healthcare, Netherlands) were rehydrated with sample in an IPGbox (GE Healthcare, Netherlands). Isoelectric focusing (IEF) was performed in the Ettan IPGphor three IEF system (GE Healthcare, Netherlands) with the following program: 0.5 h gradient to 300 V, 1 h gradient to 1,000 V, 1 h 2,000 V, 1 h 3,000 V, 1 h 4,000 V, and 1 h 5,000 V. After IEF strips were equilibrated 15 min in 3 mL equilibration buffer containing 75 mM Tris-HCl pH 8.8, 6 M Urea (Sigma Aldrich, Merck, Germany), 30% Glycerol, 2% SDS, bromophenol blue and 65 mM DTT, followed by a 15 min equilibration with equilibration buffer containing 54 mM iodoacetamide (Sigma Aldrich, Germany) instead of 65 mM DTT. Strip was placed on a 4–12% NuPAGE bis-tris Zoom gel (Thermo Fisher, Invitrogen, Netherlands) and sealed with Agarose sealing buffer (BioRad, Netherlands). Proteins were separated in MES running buffer in a Xcell surelock minicell electrophoresis system for 50 min at 200 V. Gels were stained with Coomassie or used for Western blot.

### Western Blot

Nitrocellulose membrane (Thermo Fisher, Netherlands), filters (BioRad, Netherlands), and gel were equilibrated in transfer buffer consisting of 48 mM Tris, 39 mM Glycine (Sigma Aldrich. Germany), 200 mL 96% Ethanol (VWR, Merck, Netherlands) in 1 L distilled water. Proteins were transferred from gel to membrane in 60 min, 60 mA/blot in the TE77 PWR semi-dry transfer unit (Amersham Biosciences, United Kingdom). Blot was blocked O/N at 4°C in block buffer consisting of 0.1% PBS-T pH 7.2 (PBS pH 7.2 with 0.1% w/v Tween-20), and 0.5% Protifar (Nutricia, Netherlands). Blot was incubated for 2 h, while shaking with human sera diluted 1:1,000 in block buffer, followed by three times 5 min wash with PBS-T. Subsequently, the blot was incubated with 1:5,000 diluted rabbit-anti-human IgG IR800 labeled or 1:5,000 goat-anti-human IgA IR800 labeled in block buffer and incubated for 1 h while shaking. Membranes were washed three times with 0.1% PBS-T and scanned with the Odyssey infrared imager (Licor, Westburg, Netherlands).

### Delta2D Analysis

Western blots were analyzed using Delta2D version 4.7 (Decodon, Germany). Group warping strategy was used for both IgG blots and IgA blots, meaning the four groups were linked to each other [wPV(+), wPV(–), aPV(+), aPV(–)] after the blots within the group were linked to each other. Spot detection was performed and intensities were measured in volume.

### In-gel-Digestion and Liquid Chromatography Mass Spectrometry (LC-MS) Analysis

The in-gel-digestion was performed as described previously (19). For the LC-MS analysis, samples were analyzed by nanoscale reversed-phase liquid chromatography electrospray mass spectrometry, according to the method by Meiring et al. (26). The analysis was performed on LTQ-Orbitrap XL mass spectrometer (Thermo Fisher Scientific, Germany). Analytes were loaded on a trapping column [Reprosil-Pur C18-AQ 5 μm (Dr. Maish, Germany); 23 mm long ×100 μm inner diameter] with solvent A (0.1% (v/v) formic acid in LC-MS water) in 10 min at 5 μL/min. The analytes were separated by reversed-phase chromatography on an analytical column [Reprosil-Pur C18-AQ 3 μm (Dr. Maish, Germany); 36.2 cm long ×50 μm inner diameter] at a flow rate of 100–150 nL/min. A gradient was started with solvent B [0.1% (v/v) formic acid in acetonitrile]: 7.5–57.5% in 25 min and 85% for 10 min. After the gradient, the columns were equilibrated in 100% solvent A for 10 min at 100–150 nL/min. The peptides were measured by data dependent scanning; comprising a MS-scan (m/z 300–1,500) in the orbitrap with a resolution of 60,000 (FWHM), followed by collision-induced dissociation (LTQ) of the 10 most abundant ions of the MS spectrum. The threshold value for these precursor ions was set at 1,000 counts. The normalized collision energy was set at 35% and isolation width at 2.0 Da, activation Q to 0.250 and activation time to 30 ms. The maximum ion time (dwell time) for MS scans was set to 250 ms and for MS/MS scans to 1,000 ms. Precursor ions with unknown and +1 charge states were excluded for MS/MS analysis. Dynamic exclusion was enabled (exclusion list with 500 entries) with repeat set to 1 and an exclusion duration of 15 s. The background ion at 391.28428 Da was used as lock mass for internal calibration.

Proteome Discoverer 2.1 software was used for identification of the peptide sequence data retrieved from LC-MS/MS spectra as described in Raeven et al. (19). The gel spot was assigned to one protein when intensities of this single protein was ≥70% of the intensities of all identified proteins. The spot was not assigned if the intensity of most abundant protein was <70%.

### Statistical Analysis

Data were analyzed and visualized in R statistical software (version 3.4.2). Differences in spot intensity were analyzed by one-way ANOVA. Data were visualized using Principal Component Analysis (PCA) and hierarchical clustering (Euclidean distance, Ward.D linkage). Data from antibody MIAs were log-transformed and statistically tested using a *t*-test. *P* ≤ 0.05 were considered as significant differences.

## Results

### Included Individuals

In total, sera from 36 Danish individuals were included in this study. The samples were divided in four groups (*n* = 9) (Table 1) based on a differentiation in immunization background (aPV vs. wPV) and serum anti-PTx IgG levels (– vs. +) previously measured with a diagnostic anti-PTx ELISA performed at Statens Serum Institut. Since all included individuals received their last pertussis immunization at least 10 years prior to the sampling of blood, this indicates that the antibody responses analyzed in this study are not vaccine-induced. The enhanced anti-PTx responses are therefore, an indication of a recent or ongoing *B. pertussis* infection.

### Serum Antibody Levels

A MIA was performed to determine the serum IgG antibody levels directed against PTx, Prn, FHA, and Fim2/3. Additionally, beads coupled with pertussis OMV were included to obtain semi-quantitative data on antibody responses against other antigens. Composition, identity, and concentration of the antigens, both proteins and lipopolysaccharides (LPS), of these OMVs were described previously (19).

**Figure 1:**
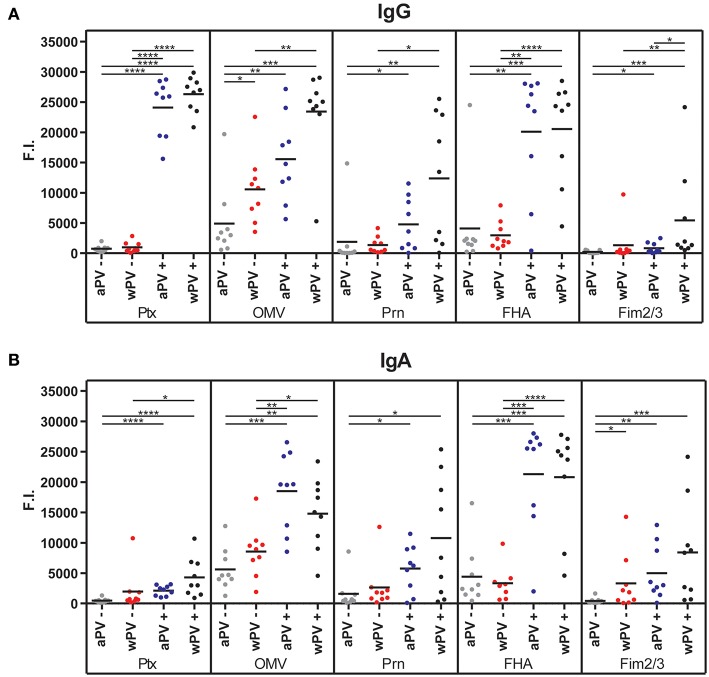
Serum IgG and IgA antibody responses. The **(A)** IgG and **(B)** IgA antibody levels against PTx, OMV, Prn, FHA, and Fim2/3 were determined in sera using a MIA. Four groups (*n* = 9) were included with a distinct immunization background (aPV, wPV) and low (–) or high (+) serum anti-PTx IgG levels. Results are depicted in fluorescence intensity (F.I.). Significant differences are indicated by ^*^p < 0.05, ^**^p < 0.01, ^***^p < 0.001, and ^****^p < 0.0001 obtained using a *t*-test after log-transformation of data.

The MIA data confirmed the significantly elevated anti-PTx IgG levels in the wPV(+) and aPV(+) groups and the absence of measurable anti-PTx IgG in the wPV(–) and aPV(–) groups (Figure 1A). Individuals in the wPV(–) and aPV(–) groups had low or absent antibody levels against FHA, Fim2/3, and Prn. However, the anti-OMV responses were elevated in all groups. The anti-OMV levels were significantly higher in the wPV(–) group as compared to the aPV(–) group and significantly higher in the high aPV(+) and wPV(+) groups as compared to their low anti-PTx antibody counterparts. The anti-Prn and anti-FHA IgG levels were, respectively, moderately and strongly enhanced in high anti-PTx groups compared to the low anti-PTx groups. A slight increase in anti-Fim2/3 IgG levels was observed in the high anti-PTx groups and these levels were significantly increased in the wPV(+) individuals as compared to the aPV(+) individuals.

The MIA data demonstrated that IgA antibody levels against PTx, OMV, Prn, FHA, and Fim2/3 were significantly enhanced in the high anti-PTx groups compared to the low anti-PTx groups (Figure 1B). No significant differences were observed between the aPV- and wPV-immunized individuals with high anti-PTx IgA levels. Whereas, anti-PTx IgA responses were absent in the low anti-PTx groups, some individuals in these groups contained low levels of anti-Prn IgA and most individuals had enhanced anti-OMV and anti-FHA IgA levels. The anti-Fim2/3 IgA was significantly higher in the wPV(–) group as compared to the aPV(–) group. In conclusion, the MIA results demonstrated that individuals with high anti-PTx IgG titers also contained high IgG and IgA antibody levels against FHA, Prn, and OMVs but no significant differences were detected between individuals vaccinated with aPV or wPV in childhood.

### IgG and IgA Responses Determined With 1-Dimensional Electrophoresis and Immunoblotting

**Figure 2:**
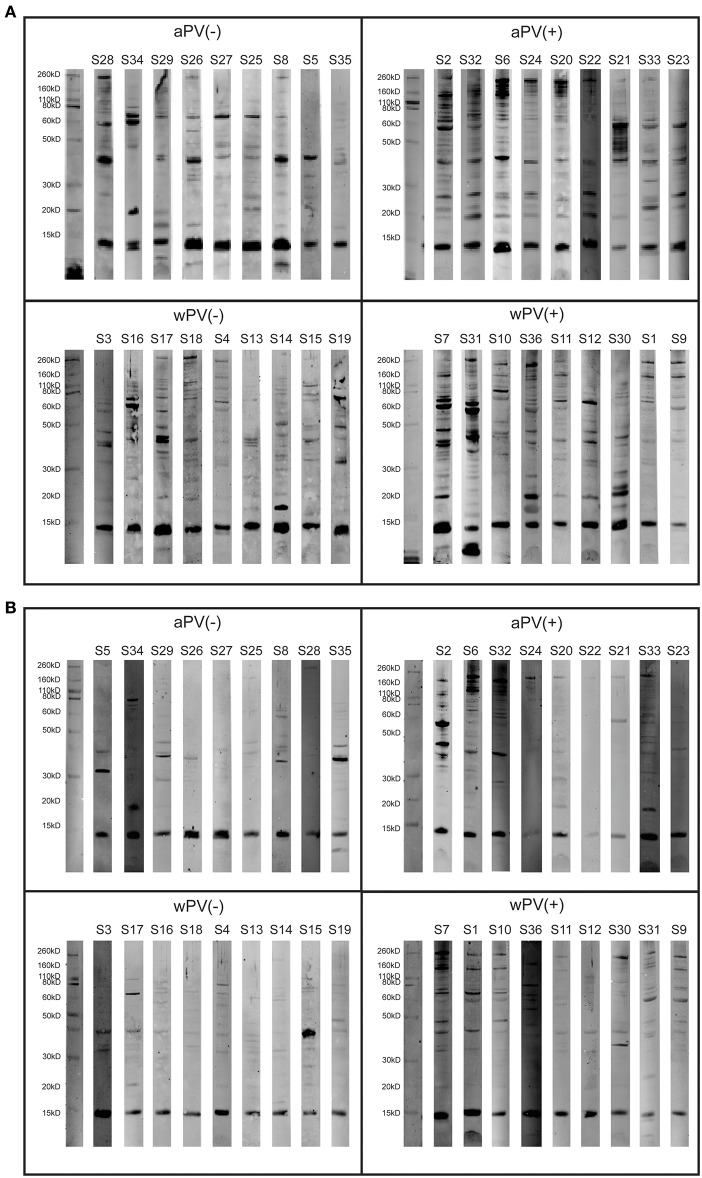
IgG and IgA responses determined with 1-Dimensional electrophoresis and immunoblotting on a *B. pertussis* B1917 lysate. **(A)** IgG and **(B)** IgA antibody profiles were analyzed in sera of 36 individuals divided over four groups (*n* = 9) with a distinct immunization background (aPV, wPV) and low (–) or high (+) serum anti-PTx IgG levels. Each group contains a marker lane (left) and nine blots representing single individual.

A combination of 1-dimensional gel electrophoresis and Western blotting (1DEWB) was used to profile the diversity in antigen-specificity of serum IgG and IgA present in the 36 individuals with different immunization background and PTx antibody-levels (Figures 2A,B). The profiling was performed by comparing the location and intensity of bands between the different individuals without quantifying these parameters.

The analysis of IgG responses demonstrated that the number and intensity of stained bands was higher in the two groups with high anti-PTx levels compared to the two groups with low anti-PTx levels (Figure 2A), which was in accordance with the higher antibody levels measured with the MIA. The presence of bands on the blots of the aPV(–) and wPV(–) group most likely corresponded with the anti-OMV antibodies that were detected with the MIA. Both the aPV(+) and wPV(+) groups contained numerous bands that were detected in multiple individuals within each group. Interestingly, this profiling demonstrated that even within a group, where individuals have the same immunization background, there was a large diversity in antibody patterns following a *B. pertussis* infection. For instance, antibody profiles of samples S2 and S32 in the aPV(+) group were similar as were the patterns of sample S33 and S23 or S24 and S20. However, when comparing the patterns of (S2/S32), (S24/S20), and (S33/S23) they revealed three distinct profiles. In the wPV(+) group the diversity was even higher as only samples S1, S9, and S11 shared a common antibody profile. Also for the serum IgA profiles the number and intensity of bands was higher in the two groups with high anti-PTx levels compared to the two groups with low anti-PTx levels (Figure 2B). Some individuals in the aPV(–) group (S5, S8, S29, S34, S35) and wPV(–) group (S3, S15, S17) showed some pertussis-specific antibody responses that were overall lower in the wPV(–) group. The comparison of responses between the aPV(+) and wPV(+) groups revealed some overlap in bands, but there was a large individual variation between and within the groups. To conclude, the 1DEWB analysis demonstrated the presence of a diverse range of antibodies in all groups but also a large variability in the response between and within groups.

### Profiling of Serum Antibody Responses Using 2-Dimensional Electrophoresis (2DE)

Proteins in a *B. pertussis* B1917 lysate were separated with 2DE (Supplementary Figure 1) to achieve better a protein separation on the gel, thereby enhancing the chance of antigen identification with LC-MS. The 2DE gels were subsequently combined with immunoblotting (2DWB). For this 2DWB analysis, a selection of serum samples were included (Table 1) for antibody profiling of IgG (4 ×4 samples) and IgA (4 ×2 samples). The selection of samples was based on the most intense signal per group within the 1DWB analysis (Figures 2A,B).

**Figure 3:**
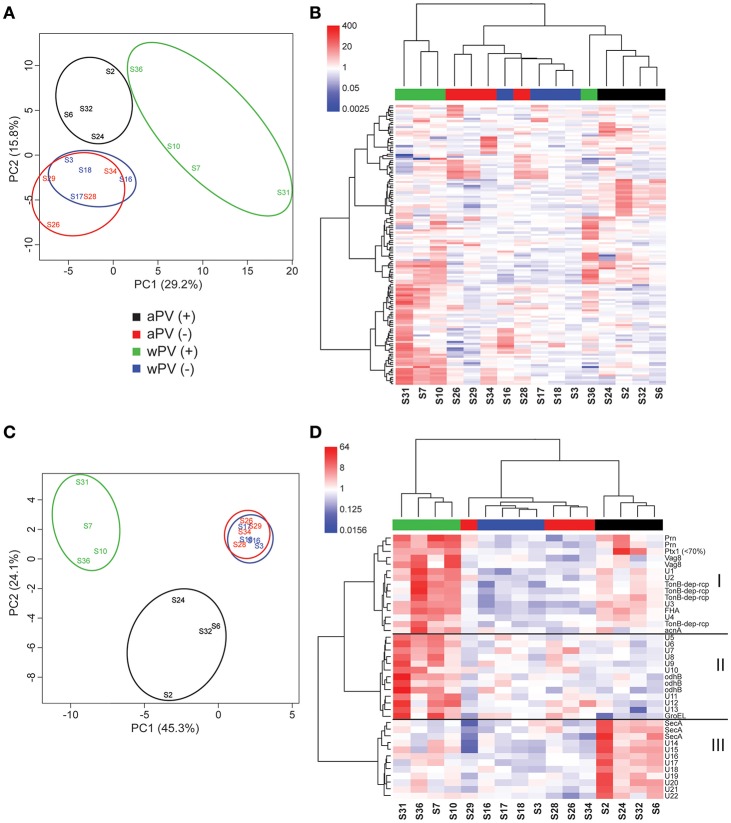
IgG responses determined with 2-Dimensional electrophoresis and immunoblotting. The presence and intensity of IgG antibodies were determined in sera for 16 individuals divided over four groups (*n* = 4). **(A)** Total variance of component 1 (PC1) and component 2 (PC2) were calculated of the total IgG responses analyzed by 2DEWB for each of the 16 individuals in the four groups. **(B)** The intensity of IgG responses of 133 spots were compared (red = higher, white = equal, blue = lower) to a common reference (median of all intensities for each individual per spot). Euclidean clustering was performed to identify patterns in antibody profiles for both the 16 individuals and the 133 spots. **(C)** Total variance of PC1 and PC2 were calculated of the significant IgG spots analyzed by 2DEWB for each of the 16 individuals in the four groups. **(D)** The intensity of the 40 spots that were found significant in the IgG response of all 16 individuals divided over four groups (*n* = 4) were compared (red = higher, white = equal, blue = lower) to a common reference (median of all intensities for each individual per spot). Euclidean clustering was performed to identify patterns in antibody profiles for both the 16 individuals and the 40 spots leading to three clusters (I–III).

For IgG, 16 sera were used for immunoblotting (Supplementary Figure 2). Using Delta2D, a warping strategy was applied to make an overlay of the 16 individual blots and to perform spot detection. This resulted in 133 unique immunogenic protein spots. Subsequently, intensities of all 133 spots were determined for each spot on each gel, which were then compared in a principle component analysis (PCA) (Figure 3A). The PCA demonstrated a similar response between aPV(–) and wPV(–) groups. Furthermore, both aPV(+) and wPV(+) groups showed more variance compared to groups with low anti-PTx levels. However, the aPV(+) group responded differently compared to the wPV(+) group. Moreover, a large variation was observed between individuals in the wPV(+) group. Especially, sample S36 of the wPV(+) group seemed to cluster together with the samples of the aPV(+) group. Next, the spot intensity of each of the 133 immunogenic proteins were compared to a common reference (the mean of the spot intensities for all 16 sera for each individual spot) and summarized in a heatmap (Figure 3B). An Euclidean clustering was performed on all 133 spots but also on the 16 sera in order to identify patterns in antibody profiles and individuals. This analysis confirmed the clustering based on the 16 sera as observed in the PCA plot where the aPV(+) and wPV(+) groups showed distinct response patterns, while sample S36 showed a large variation compared to the other samples in the wPV(+) group.

In total, 40 of the 133 spots were considered to be significant IgG responses [one-way ANOVA, false discovery rate (FDR) of 10%]. A PCA of these 40 significant immunogenic proteins revealed a similar yet even more distinct separation between the different groups as was observed for the analysis on all 133 spots (Figure 3C). The aPV(–) and wPV(–) groups clustered together indicating no difference in response. However, the aPV(+) and wPV(+) groups showed a large variance compared to the aPV(–) and wPV(–) groups but also when compared to each other. Within this analysis of 40 proteins, the variation between individuals of each group was much lower as compared to the PCA of all 133 immunogenic proteins. Based on Euclidean clustering on both the expression pattern within each individual and the intensity of the 40 spots as compared to the common reference, three different clusters (I–III) could be identified (Figure 3D). The proteins in *cluster I* showed antibody binding to both the wPV(+) and aPV(+) groups, which was higher in the wPV(+) group. The immunogenic proteins in *cluster II* did solely bind to antibodies detected in the wPV(+) group, whereas for *cluster III* this was exclusively found for samples from the aPV(+) group. Of these 40 spots, 17 spots could be identified using in-gel-digestion and LC-MS (Table 2). *Cluster I* contained well-known immunogenic pertussis antigens, such as Vag8, Prn, and FHA. However, also the TonB-dependent outer membrane receptor and the iron-dependent enzyme aconitate hydratase were identified in *cluster I*. Protein identification in *cluster II* turned out to be more challenging as only GroEL and Dihydrolipoyllysine-residue succinyltransferase component of 2-oxoglutarate dehydrogenase complex (odhB) could be successfully identified leaving nine proteins unidentified. Almost all other immunogenic spots in this cluster were found in the pI range of 3–7. The resolution of the protein separation in this region was too low to perform a reliable protein identification. Finally, the proteins in *cluster III* remained mainly unidentified, except for protein translocation subunit SecA. SecA is a protein that is involved in translocation of proteins across the inner membrane such as FHA (27). In order to enhance the chance of identifying immunogenic proteins in *cluster II* we decided to perform 2DE with an altered pI range that led to a better protein separation in the pI range 4–7 (Supplementary Figure 3A). Next, this gel was used to perform a Western blot using a single representative serum (sample S7) from the wPV(+) group as this group showed the most intense antibody binding to antigens in *cluster II* (Supplementary Figure 3B). This resulted in identification of peptidoglycan associated protein (PAL) and confirmed the identification of Dihydrolipoyllysine-residue succinyltransferase component of 2-oxoglutarate dehydrogenase complex (Supplementary Table 1). Moreover, a characteristic pattern was observed between 40 to 50 kDa and around pI 5 to 6 in samples S7, S16, S28, and S31 (Supplementary Figure 2). This pattern was also observed when analyzing sample S7 on a gel with a pI range 4–7 (Green antigens in Supplementary Figure 3B and Supplementary Table 1). LC-MS identification demonstrated that a large number of these spots contained GroEL fragments.

**Table 2:** Significant IgG responses following a *B. pertussis* infection in wPV and aPV immunized individuals.

**Cluster**	Protein^*^	**ID**	**Accesion number**	**mW (kDa)**	**pI**	**Coverage (%)**	**High confident identified peptides**
I	Pertactin autotransporter	*prn*	P14283	93.4	9.2	14.6	9
	Pertactin autotransporter	*prn*	P14283	93.4	9.2	14.6	9
	<70% confident *pertussis toxin subunit 1 based on 4-7 strip*						
	Autotransporter	*Vag8*	Q79GN7	94.8	6.8	63.7	43
	Autotransporter	*Vag8*	Q79GN7	94.8	6.8	61	38
	U1						
	U2						
	TonB-dependent outer membrane receptor	*ABC47_15525*	A0A1P9PLY0	77.8	6.6	43.6	30
	TonB-dependent outer membrane receptor	*ABC47_15525*	A0A1P9PLY0	77.8	6.6	40.4	23
	TonB-dependent outer membrane receptor	*ABC47_15525*	A0A1P9PLY0	77.8	6.6	40.4	23
	U3						
	Filamentous hemagglutinin	*FHA*	P12255	367.3	8.8	34.3	92
	U4						
	TonB-dependent outer membrane receptor	*ABC47_15525*	A0A1P9PLY0	77.8	6.6	40.4	23
	Aconitate hydratase	*acnA*	Q7VX12	97.7	7.4	30.6	26
II	U5^**^						
	U6^**^						
	U7^**^						
	U8^**^						
	U9^**^						
	U10 *putative peptidoglycan associated protein based on 4–7 strip*						
	Dsc of 2-odc^***^	*odhB*	Q7VZ17	41.8	5.5	67.6	16
	Dsc of 2-odc^***^	*odhB*	Q7VZ17	41.8	5.5	76.7	19
	Dsc of 2-odc^***^	*odhB*	Q7VZ17	41.8	5.5	67.6	16
	U11^**^						
	U12 <70% confident *Dihydrolipoyl dehydrogenase (55%), Aspartate tRNA ligase (40%)*						
	U13^**^						
	60kD chaperonin	*GroEL*	P48210	57.4	5.2	53.2	28
III	Protein translocase subunit SecA	*SecA*	Q7VUR2	103.2	5.7	52.5	36
	Protein translocase subunit SecA	*SecA*	Q7VUR2	103.2	5.7	52.5	36
	Protein translocase subunit SecA	*SecA*	Q7VUR2	103.2	5.7	52.5	36
	U14 <70% confident *Carbomoyl-phosphate synthase (35%), Aconitate hydratase B (25%)*						
	U15						
	U16						
	U17						
	U18						
	U19						
	U20						
	U21						
	U22						

* *U, Unidentified proteins*.

** *Green spots (10–24) represent a series of spots all involving the antigen GroEL*.

*** *Dihydrolipoyllysine-residue succinyltransferase component of 2-oxoglutarate dehydrogenase complex*.

**Figure 4:**
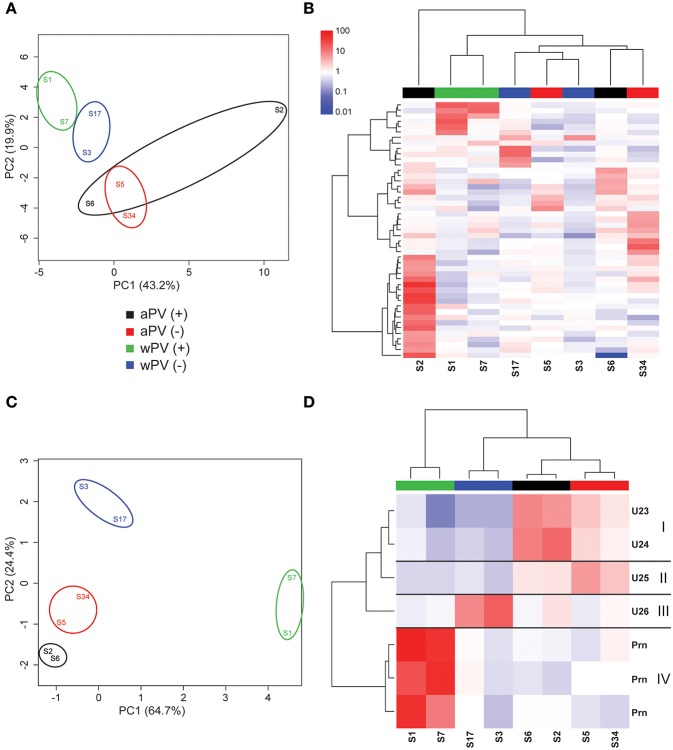
IgA responses determined with 2-Dimensional electrophoresis and immunoblotting. The presence and intensity of IgA antibodies were determined in sera for eight individuals divided over four groups (*n* = 4). **(A)** Total variance of component 1 (PC1) and component 2 (PC2) were calculated of the total IgA responses analyzed by 2DEWB for each of the eight individuals in the four groups. **(B)** The intensity of IgA responses of 47 spots were compared (red = higher, white = equal, blue = lower) to a common reference (median of all intensities for each individual per spot). Euclidean clustering was performed to identify patterns in antibody profiles for both the eight individuals and the 47 spots. **(C)** Total variance of PC1 and PC2 were calculated of the significant IgA spots analyzed by 2DEWB for each of the eight individuals in the four groups. **(D)** The intensity of the seven spots (including the unidentified proteins U23–U26) that were found significant in the IgG response of all eight individuals divided over four groups (*n* = 4) were compared (red = higher, white = equal, blue = lower) to a common reference (median of all intensities for each individual per spot). Euclidean clustering was performed to identify patterns in antibody profiles for both the 8 individuals and the seven spots leading to four clusters (I–IV).

For IgA, eight sera were used for immunoblotting (Supplementary Figure 4). The warping strategy and spot detection using Delta2D resulted in 47 unique immunogenic protein spots. As for the IgG analysis, the spot intensities of all 47 spots were obtained of each spot on each gel and subsequently compared in a PCA (Figure 4A). Here, a clustering was observed based on immunization background as the wPV groups clustered together. The aPV groups clustered together, except for sample S2. The spot intensities of the 47 immunogenic proteins were compared to a common reference and summarized in a heatmap (Figure 4B). An Euclidean clustering, performed on all 47 spots as well as the eight samples, revealed that only the wPV(+) samples S1 and S7 clustered together while the rest contained a different profile. The separate clustering of sample S2 from the rest was due to an intense cluster of spots exclusively present in this sample. Of the 47 spots, seven were considered to represent significant IgA responses (one-way ANOVA, false discovery rate (FDR) of 20%). Using these data, the aPV(+) and wPV(+) groups revealed distinct variance in the PCA (Figure 4C). Four distinct clusters (I–IV) were observed in a heatmap (Figure 4D) and unraveling the identity of the seven significant spots was attempted using LC-MS (Table 3). *Cluster I* contained two immunogenic proteins present in both aPV groups, with highest intensity in the aPV(+) group. One immunogenic protein was found in *cluster II* that showed antibody-responses in both aPV groups, but with the highest intensity in the aPV(–) group. *Cluster III* included one immunogenic protein with antibody-responses solely present in the wPV(–) group. Finally, the three immunogenic proteins of *cluster IV* were all identified as pertactin and antibody-responses were intensely and exclusively present in the wPV(+) group. All other immunogenic IgA proteins remained unidentified.

**Table 3:** Significant IgA responses following a *B. pertussis* infection in wPV and aPV immunized individuals.

**Cluster**	Protein^*^	**ID**	**Accession number**	**mW (kDa)**	**pI**	**Coverage (%)**	**High confident identified peptides**
I	U23						
	U24						
II	U25						
III	U26						
IV	Pertactin autotransporter	*Prn*	P14283	93.4	9.2	14.6	9
	Pertactin autotransporter	*Prn*	P14283	93.4	9.2	14.6	9
	Pertactin autotransporter	*Prn*	P14283	93.4	9.2	14.6	9

* *U, unidentified proteins*.

In conclusion, the 2DEWB antibody profiling revealed many previously unidentified immunogenic antigens induced by a *B. pertussis* infection. Some antigens could be identified, while others remained unidentified. A selection of these immunogenic antigens were significantly different between aPV and wPV groups and may therefore be related to the type of pertussis vaccine received in childhood.

## Discussion

The *B. pertussis* resurgence is targeting the unvaccinated population, but is also observed within the vaccinated population. In Denmark, the wPV was replaced in 1997 by a monocomponent aPV that solely contains pertussis toxoid (5). The efficacy of this aPV is high (28) and it can potentially be implemented for maternal immunization (9) making it a suitable vaccine to protect infants. However, data from recent years demonstrate that, despite a high vaccine coverage, the pertussis resurgence is also taking place in Denmark (5, 29, 30). The resurgence is however thought to be primarily due to increased diagnostic repertoire and improved awareness. The current situation provides two distinct groups in the Danish population with a different priming immunization (aPV or wPV). It was recently demonstrated that T-helper cell polarization persists following whole-cell pertussis vaccination despite repeated acellular boosters (31) Because prime immunization may also have an effect on the infection-induced antibody response, we investigated the immunoproteomic profiles in distinct groups with different vaccination backgrounds within the Danish population.

We have shown that the antibody specificity to *B. pertussis* antigens as a result of a recent pertussis infection in adolescence is correlated with the pertussis vaccine received in childhood. While the available material only allows for investigation of antibody responses, there could possibly also be differences between the aPV and wPV groups when it comes to number or type of B-cells following infection. Moreover, as the analysis of serum IgA responses on 2DEWB was only performed with two samples for each group, the power of these results is limited and only give an indication of antigens involved. As these serum IgA responses are only a reflection of the IgA responses at mucosal sites, a direct analysis of these responses in lung lavages or nasal swabs could give a better indication. The current study did however show different antibody patterns when comparing sera from individuals having received the whole-cell vaccine to sera from individuals who had received the acellular vaccine. The sera from whole-cell vaccinated individuals contained antibody responses to more different *B. pertussis* antigens. The antibody response to well-known immunogenic antigens such as FHA, Prn, and Vag8 (32) as well as other antigens such as a TonB-dependent outer membrane receptor (33) and the iron-dependent enzyme aconitate hydratase was more abundant in the wPV(+) group. Antibodies against cytosolic antigens such as GroEL and odhB was exclusively detected in the wPV(+) group. As these antigens are also present in the wPV this could resemble a recall memory response induced by vaccination. However, the discovery of antibodies to SecA (27) as well as those against ten unidentified antigens that were found exclusively in the aPV(+) group cannot be directed to a vaccine-induced memory response given that the aPV only contains pertussis toxoid. These findings may indicate that the *B. pertussis* infection is processed differently between individuals immunized with wPV or aPV in childhood leading to a distinct infection-induced antibody response. It would be interesting to determine whether immunization background also influences the severity of the disease in infected individuals. Unfortunately, additional information such as duration or severity of symptoms for these individuals was not available.

The Danish aPV is without remnants of other proteins from *B. pertussis* (34). If the aPV groups have had previous contact with other *B. pertussis* antigens than PTx, it is therefore, not originating from the vaccine. Moreover, antibody response after vaccination wane after 15 months to 2 years after immunization, to levels below detection (10, 11). A limitation to the study is that information on the infant pertussis immunizations for the wPV group was unknown. However, as the vaccination coverage in Denmark has been high throughout the whole period we assume that most of these individuals have been wPV vaccinated. The main focus, to identify if childhood aPV changes subsequent immune responses, is not hampered by this since the vaccination status of all in the aPV group is known. Also the aPV(+) and wPV(+) groups had an ongoing or recent pertussis with infection-induced anti-PTx antibodies as opposed to vaccine-induced antibodies, since (i) there are no recommendations for adolescent or adult pertussis vaccinations in Denmark, (ii) the Danish vaccination registry also contains information on adult immunizations and none was listed, and (iii) the sera were submitted for diagnostic purposes. The aPV and wPV groups differ slightly by age, as individuals in the wPV group are ~4 years older than in the aPV group. However, as all of them have grown up during the same period and in the same country, we feel confident that these groups are comparable when it comes to antibody-repertoire.

We have demonstrated that the antibody-response during *B. pertussis* infection is not limited to the antigens contained in the childhood vaccine, since individuals who had been vaccinated with a monocomponent pertussis toxoid vaccine in childhood showed increased IgG and IgA responses to OMV, Prn, FHA, and Fim2/3 at ongoing pertussis. Two previous papers have claimed that due to vaccination background, the responses to a *B. pertussis* infection are limited to the antigens present in the vaccine (13, 14). However, these studies described infections occurring in the follow-up time of clinical trials, respectively, 15 months (35), 2 years (36), 3.5 years (37), and ~3 years (13). This indicates that for all the individuals in these older studies, the *B. pertussis* infection occurred within the timeframe in which the vaccine-induced immunity should not have waned fully. Those cases could therefore be regarded as “vaccine failures.” On the other hand, the pertussis cases in the present study occurred more than 9 years after the last pertussis vaccination, so the protective effect of the vaccine-induced immunity is expected to be low or to have completely waned. The difference between the observations between the previous and present study could possibly be a difference in the immune-response between individuals in which the vaccine might have failed to induce a proper immune response and individuals where the vaccine protected as intended. The question is whether there in fact was a “linked-epitope suppression” or “original antigenic sin effect” in the previous studies or if the observations were caused by other factors. The simplest explanation can be the difference in response between first or second encounter with an antigen: the individuals with vaccine-failures in the older studies only showed brisk secondary immune-responses to the previously recognized vaccine-antigens, while responses to other antigens gave a less pronounced primary response. A different explanation to the older observations could also be an underlying immunological defect in some of the children leading to the actual vaccine failure and therefore an aberrant immune response. For one of the previous studies, the comparison of antibodies at ongoing pertussis were made between 8 and 16 year old unvaccinated children and 13–36 months old children that got infected though they were vaccinated 2 years, or less, before, with the older children showing the highest antibody-responses. The observed differences in that study could therefore be a simple effect of different age, and in particular that the older children in the study had most likely met cross-reacting antigens while growing up, thus giving rise to a strong secondary response at infection compared to the younger children that got infected despite being recently vaccinated.

The applied immunoproteomic profiling has become an important tool to determine antibody responses in pertussis research as was demonstrated for responses after pertussis infection (15, 19, 25, 38, 39) and after immunization with different pertussis vaccines (19–21). Previous studies that have implemented 2DE for pertussis research used either Tohama, Saadet (38, 40), the Chinese WCV strain 58003 (21) or varied with the antigen content as a result of iron starvation (41). Prior to the current study it was unfortunately not possible to retrieve information about which *B. pertussis* strain(s) infected the individuals included in this study. It is important to realize that the use of a different strain, and more importantly, different growth conditions could affect the antigen composition of the lysate used for Western blotting. This could have an influence on the outcome of the results of antibody profiling. In this study we included the *B. pertussis* B1917 strain, a Dutch clinical isolate from 2000 that is closely related to the current circulating strains globally (42) and the growth conditions were chosen to maximize the expression of virulence factors. The antigen composition of this lysate has been characterized in detail before, confirming the virulent state of the bacteria (19). While this strategy reduces the risk of a mismatch between the antigen composition of the lysate and the antibodies in the serum to a minimum, this method cannot guarantee that some potentially immunogenic antigens were missed. For a few of the individuals in the groups with low anti-PTx antibodies (wPV– and aPV–), an elevated antibody response to OMV, Prn, FHA, or Fim2/3 was observed, sometimes in the same individual. Given the large variance in these antibodies, the high levels of antibodies are caused by either (i) a previous *B. pertussis* infection where anti-PTx antibodies have waned, (ii) cross-reacting antibodies from other recent or ongoing infections with other *Bordetellae* (43), or (iii) cross-reacting antibodies from recent or ongoing infections with bacteria who display surface proteins similar to *B. pertussis* such as has been demonstrated for FHA (44, 45). This pattern emphasizes the relevance of recommendations for using solely anti-PTx serology for diagnostic purposes of *B. pertussis* due to the high specificity of these antibodies (46). For future clinical studies involving pertussis vaccines or a *B. pertussis* human challenge model (47) it is crucial to investigate pre-existing antibody profiles as these can influence the outcome of such studies. For instance in the clinical phase I study investigating the live-attenuated pertussis vaccine BPZE1, investigators used anti-PTx IgG levels as pre-screening parameters to exclude individuals. Afterwards, high pre-existing anti-Prn IgG levels were found to be influencing the required colonization of the vaccine and therefore, the efficacy (48). This demonstrates the requirement of a complete profiling of individuals within clinical studies to prevent confounding of results. The current method could be used to obtain these profiles and therefore providing a valuable tool for pertussis research.

In conclusion, this antibody profiling approach with 2D gel electrophoresis allowed to differentiate *B. pertussis* infection-induced antibody responses in adolescents and young adults with distinct childhood immunization background. Importantly, this study showed that the antibody responses in individuals immunized with a monocomponent pertussis toxoid only aPV in childhood were not limited to anti-PTx alone. Overall, we demonstrated that the type of childhood pertussis immunization give rise to distinct antibody responses following a *B. pertussis* infection later in life. Future studies could focus on whether this difference in response could also be related to severity of disease or particular symptoms as a result of the different immunization background.

## Data Availability

All datasets generated for this study are included in the manuscript and/or the Supplementary Files.

## Ethics Statement

Left-over material of serum submitted for diagnostic analysis in the period 2014–2016 at Statens Serum Institut, Copenhagen, Denmark was used. The sera had all been submitted for diagnosis of pertussis by analysis of anti-PTx IgG. Information on the sera is shown in Table 1. The study was approved by the National Committee on Health Research Ethics, Denmark (H-16037386).

## Author Contributions

RR, LvdM, BM, GK, and TD conceived the experiments. RR and LvdM conducted the experiments. RR, LvdM, and JP analyzed the results. RR, LvdM, EvR, GK, and TD wrote the main manuscript. JP, KF, CJ, and BM reviewed the manuscript.

### Conflict of Interest Statement

RR, LvdM, EvR, BM, and GK were employed by Intravacc. Intravacc was, at the time of submission of the manuscript, completely funded by the Dutch Government. The remaining authors declare that the research was conducted in the absence of any commercial or financial relationships that could be construed as a potential conflict of interest.

## Supplementary Material

Supplementary Figure 1 Coomassie staining of *B. pertussis* B1917 bacterial lysate separated with 2DE. Coomassie staining of *B. pertussis* B1917 separated on a 2DE gel with a pI 3–10 NL range.

Supplementary Figure 2 IgG responses determined with 2-Dimensional electrophoresis and immunoblotting. Sera of 16 selected individuals divided over four groups (*n* = 4) with a distinct immunization background (aPV, wPV) and low (–) or high (+) serum anti-PTx IgG levels were analyzed for IgG antibody profiles on a *B. pertussis* B1917 lysate separated with 2DE. Each blot represents one individual and contains a size marker (left).

Supplementary Figure 3 *Bordetella pertussis* B1917 antigen profile on a pI 4–7 range and IgG immunoproteomic profile. **(A)** Coomassie staining of *B. pertussis* B1917 separated on a 2DE gel with a pI 4–7 range. This gel was used for **(B)** Western blotting with serum of sample S7 leading to a immunoproteomic profile including two known antigens (GroEL, Prn) and 36 unknown antigens that were further identified with LC-MS as shown in Supplementary Table 1. Green spots represent a series of spots involving GroEL.

Supplementary Figure 4 IgA responses determined with 2-Dimensional electrophoresis and immunoblotting. Sera of eight selected individuals divided over four groups (*n* = 2) with a distinct immunization background (aPV, wPV) and low (–) or high (+) serum anti-PTx IgG levels were analyzed for IgA antibody profiles on a *B. pertussis* B1917 lysate separated with 2DE. Each blot represents one individual and contains a size marker (left).

Supplementary Table 1 IgG response on pI 4-7 gel.
