# Supplementary figures and images for: Antibody Specificity Following a Recent Bordetella pertussis Infection in Adolescence Is Correlated With the Pertussis Vaccine Received in Childhood
Source: Front Immunol. 2019 Jun 17;10:1364. doi: 10.3389/fimmu.2019.01364 (PMC6592373; doi:10.3389/fimmu.2019.01364)

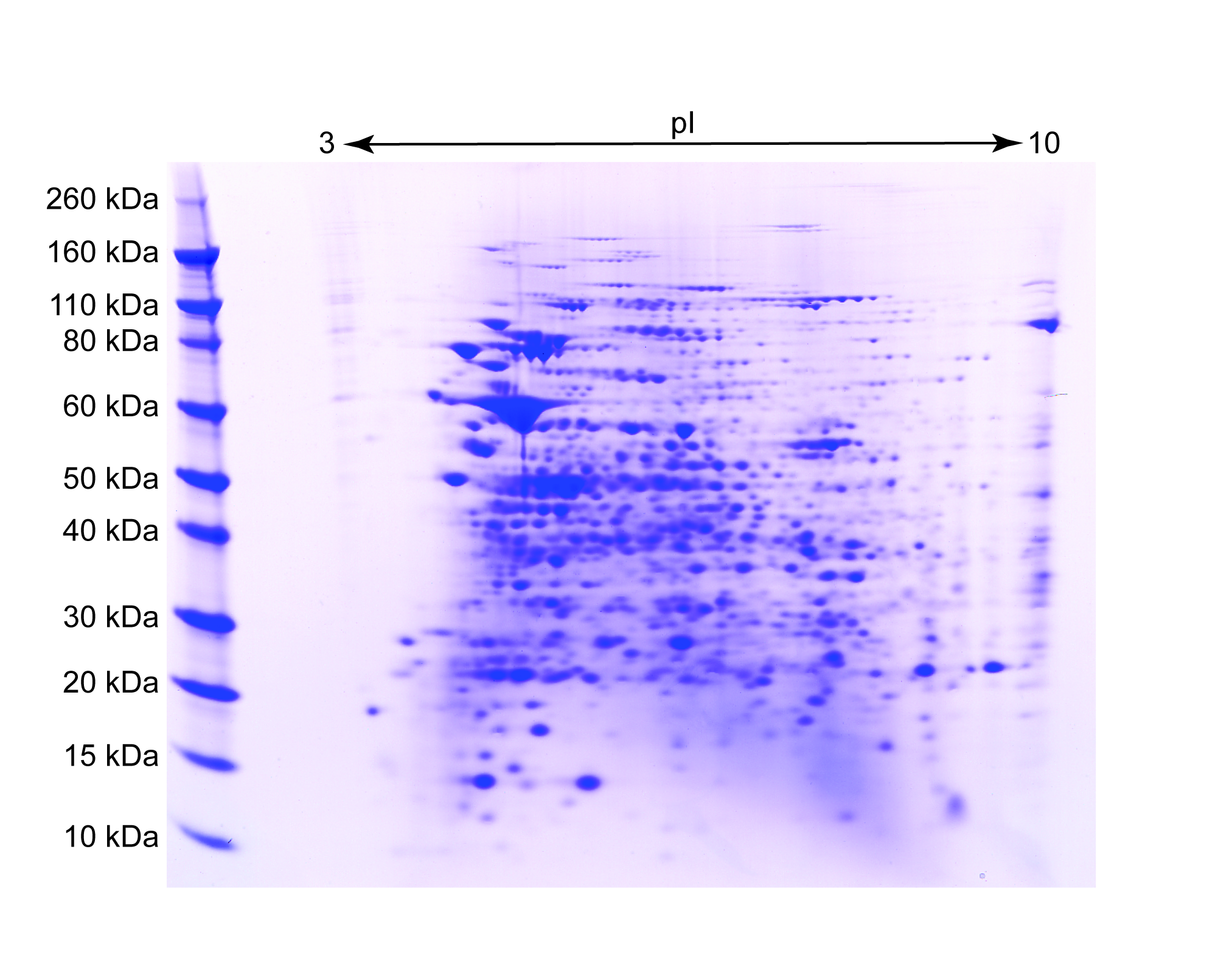

Supplement: Supplementary Figure 1 — Coomassie staining of B. pertussis B1917 bacterial lysate separated with 2DE. Coomassie staining of B. pertussis B1917 separated on a 2DE gel with a pI 3–10 NL range. [file Image_1.TIF]

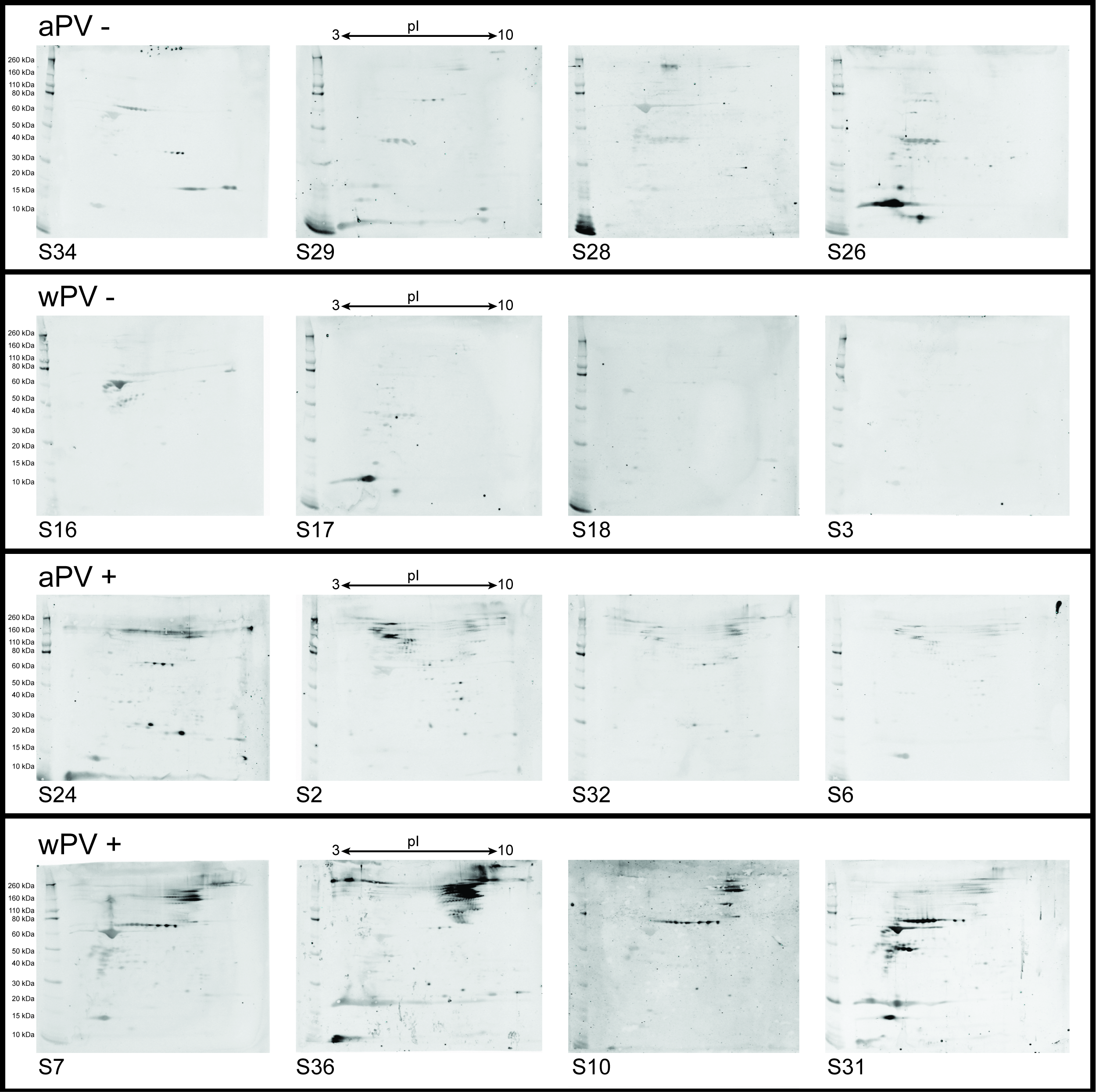

Supplement: Supplementary Figure 2 — IgG responses determined with 2-Dimensional electrophoresis and immunoblotting. Sera of 16 selected individuals divided over four groups (n = 4) with a distinct immunization background (aPV, wPV) and low (–) or high (+) serum anti-PTx IgG levels were analyzed for IgG antibody profiles on a B. pertussis B1917 lysate separated with 2DE. Each blot represents one individual and contains a size marker (left). [file Image_2.TIF]

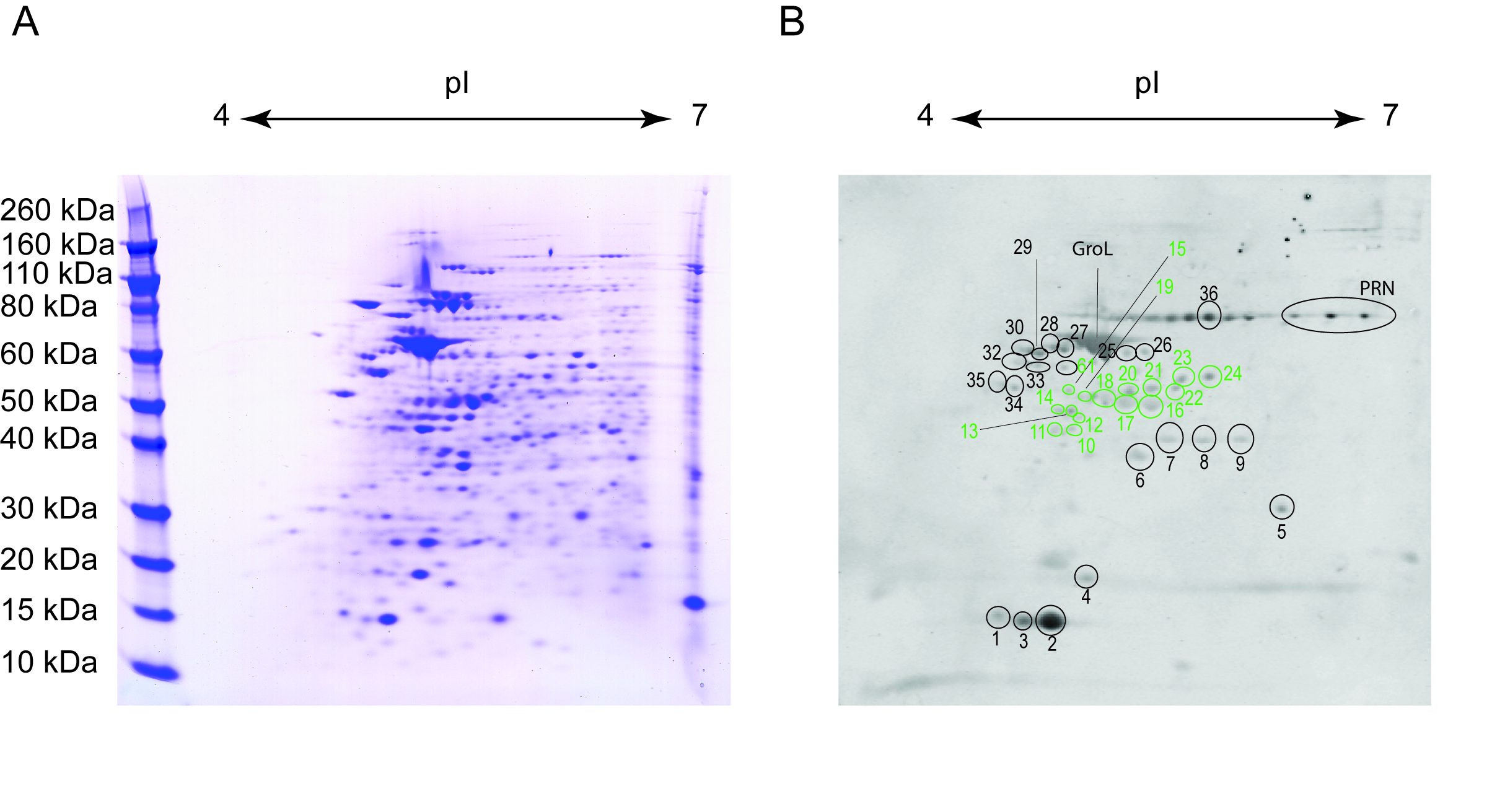

Supplement: Supplementary Figure 3 — Bordetella pertussis B1917 antigen profile on a pI 4–7 range and IgG immunoproteomic profile. (A) Coomassie staining of B. pertussis B1917 separated on a 2DE gel with a pI 4–7 range. This gel was used for (B) Western blotting with serum of sample S7 leading to a immunoproteomic profile including two known antigens (GroEL, Prn) and 36 unknown antigens that were further identified with LC-MS as shown in Supplementary Table 1. Green spots represent a series of spots involving GroEL. [file Image_3.TIF]

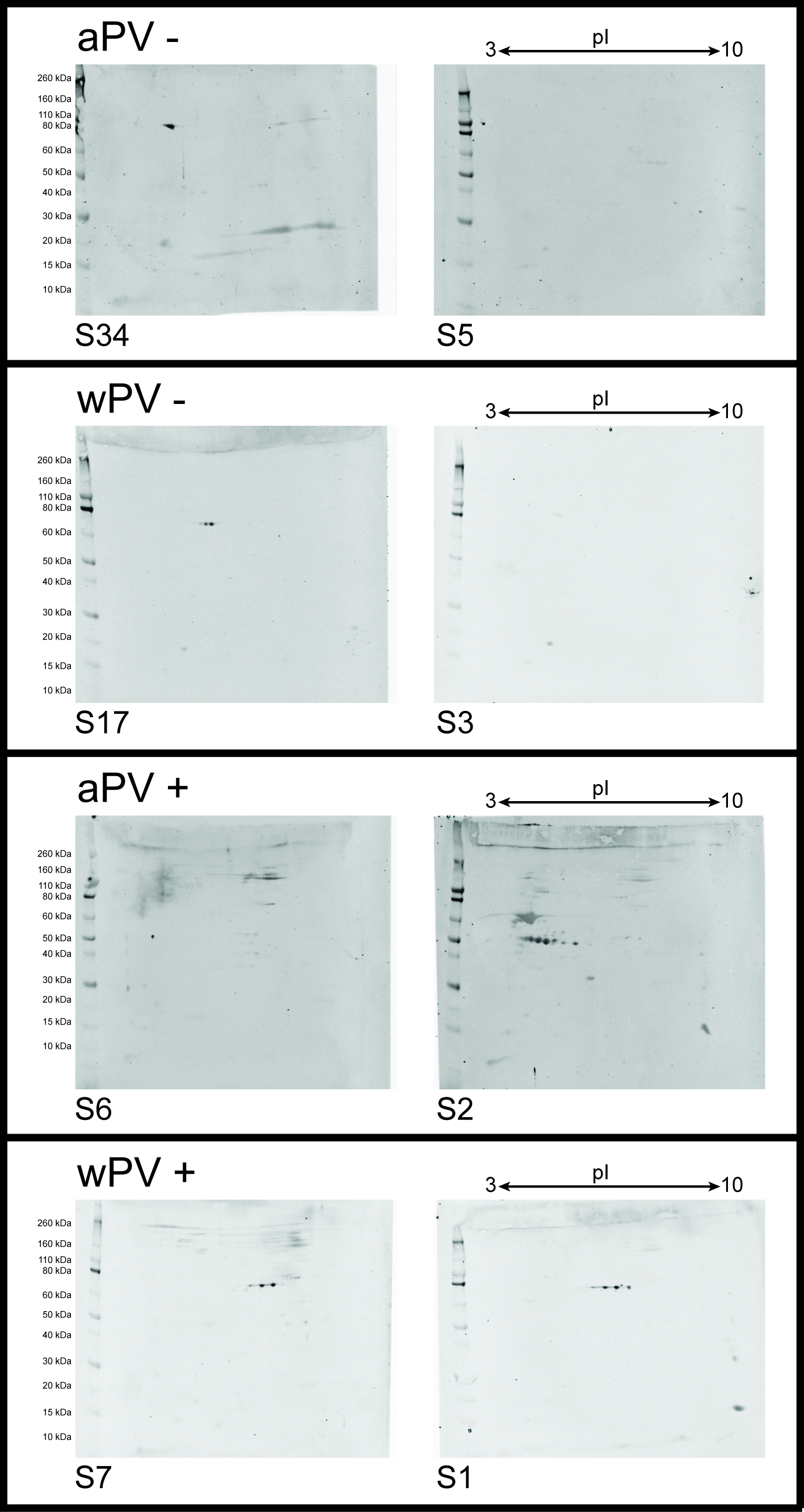

Supplement: Supplementary Figure 4 — IgA responses determined with 2-Dimensional electrophoresis and immunoblotting. Sera of eight selected individuals divided over four groups (n = 2) with a distinct immunization background (aPV, wPV) and low (–) or high (+) serum anti-PTx IgG levels were analyzed for IgA antibody profiles on a B. pertussis B1917 lysate separated with 2DE. Each blot represents one individual and contains a size marker (left). [file Image_4.TIF]
