# Supplementary material for: Antibody Specificity Following a Recent Bordetella pertussis Infection in Adolescence Is Correlated With the Pertussis Vaccine Received in Childhood
Source: Front Immunol. 2019 Jun 17;10:1364. doi: 10.3389/fimmu.2019.01364 (PMC6592373; doi:10.3389/fimmu.2019.01364)
Supplement: Supplementary Table 1 — IgG response on pI 4-7 gel. [file Table_1.DOCX]

**Supplementray Table 1 – IgG response on pI 4-7 gel**

| **Sample number** | **Protein** | **ID** | **Accesion number** | **mW (kDa)** | **pI** | **Coverage (%)** | **High confident identified peptides** |
| --- | --- | --- | --- | --- | --- | --- | --- |
| **1** | **NlpE N-terminal domain protein** | *Az26_2572* | A0A171JY90 | 15.6 | 5.45 | 51.4 | 5 |
| **2** | **50S ribosomal protein L7/L12** | *rplL* | Q7W0S0 | 12.8 | 4.9 | 34.6 | 4 |
| **3** | **50S ribosomal protein L7/L12** | *rplL* | Q7W0S0 | 12.8 | 4.9 | 28.3 | 3 |
| **4** | **Putative peptidoglycan-associated lipoprotein** | *BP3342* | Q7VU04 | 17.9 | 8.1 | 17.9 | 13 |
| **5** | **<70% confident** *Pertussis toxin subunit 1(50%), antioxidant protein (30%)* |  |  |  |  |  |  |
| **6** | **Elongation factor Ts** | *tsf* | Q7VYC9 | 30.9 | 5.4 | 67.5 | 16 |
| **7** | **Putative L-lactate dehydrogenase** | *Idh* | Q7VSM5 | 37.2 | 6 | 31.9 | 10 |
| **8** | **Putative L-lactate dehydrogenase** | *Idh* | Q7VSM5 | 37.2 | 6 | 31.6 | 9 |
| **9** | **<70% confident** *Putative L-lactate dehydrogenase (55%), DNA-directed RNA polymerase subunit alpha (30%)* |  |  |  |  |  |  |
| **10**** | **<70% confident** *60 kDa chaperonin (60%), outer membrane porin protein BP0840 (20%)* |  |  |  |  |  |  |
| **11** | **<70% confident** *outer membrane porin protein BP0840 (45%), 60 kDa chaperonin (40%)* |  |  |  |  |  |  |
| **12** | **<70% confident** *60 kDa chaperonin (40%), phosphoglycerate kinase (35%)* |  |  |  |  |  |  |
| **13** | **60kDa chaperonin** | *groEL* | P48210 | 57.4 | 5.2 | 42.2 | 23 |
| **14** | **<70% confident** *60 kDa chaperonin (60%), phosphoglycerate kinase (20%)* |  |  |  |  |  |  |
| **15** | **<70% confident** *60 kDa chaperonin (45%), Dsc of 2-odc* (25%)* |  |  |  |  |  |  |
| **16** | **Dsc of 2-odc*** | *odhb* | Q7VZ17 | 41.8 | 5.5 | 55.4 | 17 |
| **17** | **Dsc of 2-odc*** | *odhb* | Q7VZ17 | 41.8 | 5.5 | 36.6 | 13 |
| **18** | **<70% confident** *Dsc of 2-odc* (40%), 60 kDa chaperonin (40%)* |  |  |  |  |  |  |
| **19** | **<70% confident** *60 kDa chaperonin (45%), cell division protein FtsZ (20%)* |  |  |  |  |  |  |
| **20** | **<70% confident** *Aspartinokinase (65%), 60 kDa chaperonin (25%)* |  |  |  |  |  |  |
| **21** | **<70% confident** *60 kDa chaperonin (55%), ATP-dependent Clp Protease ATP-binding subunit ClpX (25%)* |  |  |  |  |  |  |
| **22** | **No proteins found** |  |  |  |  |  |  |
| **23** | **60kDa chaperonin** | *groEL* | P48210 | 57.4 | 5.2 | 42.4 | 23 |
| **24** | **60kDa chaperonin** | *groEL* | P48210 | 57.4 | 5.2 | 42.6 | 23 |
| **25** | **<70% confident** *60 kDa chaperonin (50%), Glutamine synthetase (30%)* |  |  |  |  |  |  |
| **26** | **60kDa chaperonin** | *groEL* | P48210 | 57.4 | 5.2 | 41.1 | 24 |
| **27** | **60kDa chaperonin** | *groEL* | P48210 | 57.4 | 5.2 | 40.2 | 24 |
| **28** | **60kDa chaperonin** | *groEL* | P48210 | 57.4 | 5.2 | 35.5 | 20 |
| **29** | **60kDa chaperonin** | *groEL* | P48210 | 57.4 | 5.2 | 51.9 | 27 |
| **30** | **60kDa chaperonin** | *groEL* | P48210 | 57.4 | 5.2 | 44.4 | 24 |
| **31** | **<70% confident** *60 kDa chaperonin (50%), Aspartyl/glutamyl-tRNA amidotransferase subunit B* |  |  |  |  |  |  |
| **32** | **60kDa chaperonin** | *groEL* | P48210 | 57.4 | 5.2 | 34 | 18 |
| **33** | **<70% confident** *Trigger factor (60%), 60 kDa Chaperonin (30%)* |  |  |  |  |  |  |
| **34** | **Enolase** | *eno* | Q7VW79 | 45.9 | 4.8 | 28.7 | 12 |
| **35** | **Enolase** | *eno* | Q7VW79 | 45.9 | 4.8 | 28.7 | 12 |
| **36** | **<70% confident** *Acetyltransferase component of pyruvate dehydrogenase complex (40%), putative peptidase (25%)* |  |  |  |  |  |  |

* Dihydrolipoyllysine-residue succinyltransferase component of 2-oxoglutarate dehydrogenase complex

** Green spots (10-24) represent a series of spots all involving the antigen GroEL.
